# Maternal pregnancy-related infections and autism spectrum disorder—the genetic perspective

**DOI:** 10.1038/s41398-022-02068-9

**Published:** 2022-08-16

**Authors:** Ron Nudel, Wesley K. Thompson, Anders D. Børglum, David M. Hougaard, Preben B. Mortensen, Thomas Werge, Merete Nordentoft, Michael E. Benros

**Affiliations:** 1 CORE-Copenhagen Research Centre for Mental Health, Mental Health Centre Copenhagen, Copenhagen University Hospital, Copenhagen, Denmark; 2 iPSYCH, The Lundbeck Foundation Initiative for Integrative Psychiatric Research, Aarhus, Denmark; 3 Institute of Biological Psychiatry, Mental Health Centre Sct. Hans, Mental Health Services Copenhagen, Roskilde, Denmark; 4 Department of Family Medicine and Public Health, Division of Biostatistics, University of California, San Diego, CA USA; 5 Department of Biomedicine, Aarhus University and Centre for Integrative Sequencing, iSEQ, Aarhus, Denmark; 6 Aarhus Genome Center, Aarhus, Denmark; 7 Center for Neonatal Screening, Department for Congenital Disorders, Statens Serum Institut, Copenhagen, Denmark; 8 National Center for Register-Based Research, Aarhus University, Aarhus, Denmark; 9 Department of Clinical Medicine, Faculty of Health and Medical Sciences, University of Copenhagen, Copenhagen, Denmark; 10 Department of Immunology and Microbiology, Faculty of Health and Medical Sciences, University of Copenhagen, Copenhagen, Denmark

**Keywords:** Autism spectrum disorders, Genomics

## Abstract

Autism spectrum disorder (ASD) refers to a group of neurodevelopmental disorders which include deficits in behavior, social interaction and communication. ASD has a complex genetic architecture, and it is also influenced by certain environmental exposures. Both types of predisposing factors may be related to immunological mechanisms, involving, for example, immune system genes and infections. Past studies have shown an association between infections occurring during the pregnancy in the mother and increased risk of ASD in the child, an observation which has received recent support from experimental animal studies of ASD-like behavior. The aim of this study was to study the genetic contribution to this effect. We employed genetic correlation analyses across potential ASD subtypes stratified on the basis of maternal pregnancy-related infections within the iPSYCH ASD case-cohort sample, as well as a case-case GWAS. We validated the trends of the genetic correlation analyses observed in our sample using GWAS summary statistics from the PGC ASD study (excluding iPSYCH). The genetic correlation between ASD with a history of maternal pregnancy-related infections and ASD without a history of maternal infections in iPSYCH was *r*_g_ = 0.3811. We obtained a similar estimate between the former and the PGC ASD phenotype (*r*_g_ = 0.3997). Both estimates are lower compared to the genetic correlation between ASD without a history of maternal infections and the PGC ASD phenotype (*r*_g_ = 0.6735), and between ASD with a history of maternal infections occurring only more than 2 months following childbirth and the PGC ASD phenotype (*r*_g_ = 0.6293). Additionally, we observed genetic variance between the two main ASD phenotypes using summary statistics from the case-case GWAS in iPSYCH (*h*^2^_cc_ = 0.1059), indicating genome-wide differences between the phenotypes. Our results suggest potentially different etiologies of ASD based on a history of maternal pregnancy-related infections, which may, in part, be genetic. This highlights the relevance of maternal pregnancy-related infections to genetic studies of ASD and provides new insights into the molecular underpinnings of ASD.

## Introduction

Autism spectrum disorder (ASD) includes a group of neurodevelopmental disorders which involve deficits in the domains of behavior, social interaction and communication [1]. The etiologies of the various types of ASD are heterogeneous, involving both genetic and environmental factors [2], and the former include both common and rare variants [3, 4]. Family-based estimates of ASD heritability tend to be high, e.g., 83%, or 69% for additive effects [5], whereas estimates from genetic markers in unrelated individuals tend to be lower, e.g. around 10% [3, 6]. ASD is also associated with environmental exposures such as infections, and associations between immune system genes and ASD have been reported [7–13]. Environmental factors that have been shown to be associated with risk of ASD in the child include maternal infection and/or inflammation during the pregnancy [14]. One study examined Danish nationwide register data and found a positive association between maternal bacterial and viral infections requiring hospitalization during specific stages of the pregnancy and risk of ASD in the child [15]. Moreover, it has also been shown that infections in the very early days of life are also associated with a higher risk of ASD [16]. A recent large Swedish register-based study confirmed this link, even after adjusting for many possible confounding factors such as birth order, birth seasons, gestational age, and delivery-related factors [17]. Interestingly, animal models have also provided evidence for a connection between maternal immune activation during pregnancy and ASD-like behavior in the offspring [18–21]. In a previous study of the Danish health register data we observed both a strong genetic correlation and, in a random population sample, a strong epidemiological correlation between overall infection and overall psychiatric disorder [7]. However, while both the epidemiological and the genetic risks of co-occurring infections and psychiatric disorders have been assessed at a one-generational level (i.e. when the infection and psychiatric diagnoses are assessed in unrelated individuals), the effect of maternal pregnancy-related infections on the psychiatric outcome in the child has only been studied from the epidemiological perspective, and genetic studies, in this context, are lacking. In particular, there could be several potential mechanisms through which the risk of ASD increases following maternal infection during the pregnancy or in early development: it could be that this effect is mostly environmental, but it could also be the case that the child’s genetics may play a part e.g., in how the child responds to the infection in the mother during development. Even if the effect is a direct result of the infection in the mother, it could suggest that there are different types of genetic predisposition to developing ASD, whereby some children have enough genetic risk variants to push them over the liability threshold for ASD, while other children have fewer risk variants, but maternal infection during early development, in combination with any other risk factors such as genetics, could result in ASD. In this scenario, one could think of different etiological subtypes of ASD with different genetic backgrounds.

**Fig. 1:**
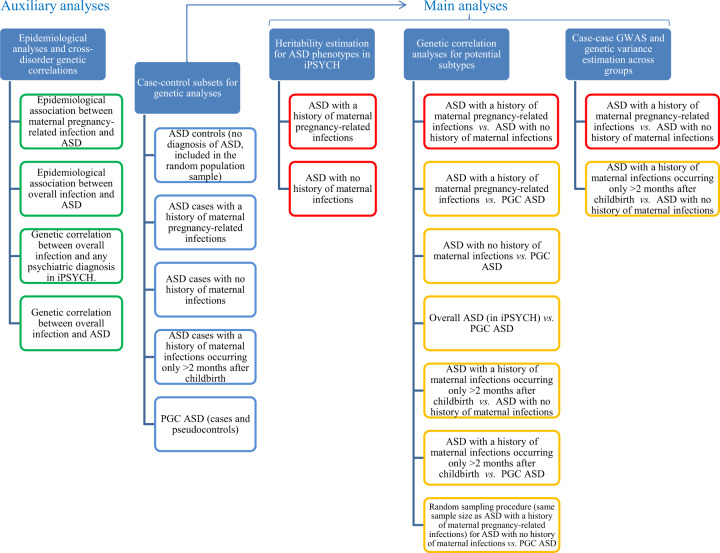
Overview of the analyses in the current study. Green boxes refer to auxiliary analyses (providing context for the main analyses and/or replicating previous findings); blue boxes refer to definitions of cases and controls for the main genetic analyses; red boxes refer to primary genetic analyses; lastly, orange boxes refer to secondary genetic (sensitivity) analyses, including analyses with internal control phenotypes and replication.

In this study we examined the relevance to the study of the genetics of ASD of stratifying ASD cases based on exposure to maternal infections requiring hospitalization/hospital contact (hereafter: infections) during or shortly after the pregnancy. We defined the relevant period for maternal infections as the period of 9 months prior to the date of birth of the offspring and up to 2 months after it. This afforded us a “safety margin” that covered maternal infections which started before childbirth but were diagnosed after it, as well as cases in which the infection and/or inflammation cytokines might have passed from the mother to the child during the puerperium, for example through breastfeeding [22, 23]. Furthermore, many of the pregnancy-related International Classification of Diseases (ICD) infection diagnoses used in our studies (Supplementary Table S1) encompass both the period of the pregnancy itself and the puerperium (for reasons of economy, we refer to any infection diagnosis given in this period as pregnancy-related). We tested this range using both epidemiological data and an internal genetic control. We used both genetic correlation analyses and a case-case genome-wide association study (GWAS) design to explore the similarities and differences between potential ASD subtypes stratified based on a history of maternal pregnancy-related infections and confirmed our findings using an independent sample. An overview of the different analyses in this study is shown in Fig. 1.

## Methods

### Data sources for diagnoses and study sample

Data were obtained by linking Danish population-based registers using the unique personal identification number employed in Denmark since 1968 [24]. The Danish Neonatal Screening Biobank stores dried blood spots taken 4–7 days after birth from nearly all infants born in Denmark after 1981 [24, 25]. Information about infections was obtained from the Danish National Hospital Register, which, since 1977, contains records of all inpatients treated in Danish non-psychiatric hospitals, and, since 1995, contains information regarding outpatient and emergency room contacts [26]. The Psychiatric Central Research Register covers all psychiatric inpatient facilities since 1969 and outpatient contacts since 1995 [27]. Diagnostic information was based on the 8th revision of the International Classification of Diseases (ICD-8) [28] from 1977 to 1993, and the 10th revision (ICD-10) from 1994 [29]. For psychiatric disorders, diagnoses from the Psychiatric Central Research Register for psychiatric inpatient, outpatient and emergency care unit contact were included. For infections, the included types of diagnosis (from the Danish National Hospital Register) were the following: ICD-8: hoveddiagnose and bidiagnose (main and auxiliary diagnosis, respectively); ICD-10: aktionsdiagnose, grundmorbus, bidiagnose (main, basic and auxiliary diagnosis, respectively). Tillægsdiagnoser (associated diagnoses) were not considered. Diagnoses of the following types were excluded: henvisningsdiagnose (referral), komplikation (complication), as were ICD-8 diagnoses with the following modifications: “Obs. Pro” and “Ej befundet” (suspected and not found, respectively). The types of contact included inpatient and outpatient hospitalizations and emergency room contact. The individuals in this study are part of the iPSYCH 2012 cohort [30], nested within all individuals in the Danish population born between 1981 and 2005 (*N* = 1,472,762), and which included individuals diagnosed with at least one of: schizophrenia, bipolar disorder or depression (affective disorder), autism spectrum disorder, attention deficit/hyperactivity disorder and anorexia, or included as part of a random population sample. Data pertaining to hospitalization for infections for iPSYCH individuals and their parents were obtained from the National Hospital Register. The iPSYCH sample has undergone extensive quality control (QC), as described in our previous studies [7, 8, 31]. Importantly, individuals were removed based on ancestry (if they did not have Danish ancestry, as determined from registry data of family history and two rounds of genetic principal component analysis; a third one was performed in the QCed sample to generate principal components (PCs) for use as covariates) and relatedness (if they were first or second degree relatives of other individuals in the sample, based on genetic analysis, prioritizing first iPSYCH cases and then individuals with a higher genotype call rate). Individuals were also removed based on missingness (1%), abnormal heterozygosity, ambiguous sex (based on genetic markers), or if they were duplicates of other individuals. The first study employing this QC protocol has more information about the procedures [6]. Before QC, we had genotypes for 78,050 individuals. Following genetic and record-based QC, 65,534 unrelated Danish individuals were retained for downstream analyses.

### Psychiatric diagnoses and infection diagnoses in our study

Data for infection diagnoses for each individual and, where possible, their parents were up to the end of 2012, and the data for the psychiatric diagnoses were up to the end of 2013. The focus of this study was maternal infections requiring hospitalization/hospital contact that occurred shortly before, during or shortly after the pregnancy (i.e., we generated a variable for the iPSYCH individual for a maternal infection, which would be 1, if their mother had a pregnancy-related infection, or 0, if the mother had no medical history of infections) and their effect on a diagnosis of ASD in the child (ICD-10 codes: F84.0, F84.1, F84.5, F84.8, F84.9). As in our previous studies, binary outcomes were coded as 1 (case) or 0 (control) for epidemiological/statistical analyses, and 2 (case) and 1 (control) for genetic analyses (the GWASs). The infection categories used in this study were as follows: bacterial, viral, central nervous system infection, gastrointestinal, genital, hepatitis, otitis, pregnancy infection, respiratory, sepsis, skin infection, HIV/AIDS, urological and other infections (e.g., protozoan infections). ICD codes for these are provided in Supplementary Table S1. There were 12,331 individuals with a diagnosis of ASD in our QCed sample of 65,534 individuals. We treated infections occurring in the period from 9 months prior to the date of birth of the iPSYCH individual to 2 months after the birth as occurring “within the time range” relevant to this study. These infections were considered “pregnancy-related” infections for the purpose of this study. Other maternal infections were considered “outside the range”. Infections occurring only more than 2 months following childbirth were used in defining an internal control for the genetic analyses as explained later. Individuals with a history of maternal infections occurring only earlier than 9 months prior to the birth were excluded from being ASD cases in the genetic analyses (except for in analyses in which all ASD cases were used irrespective of maternal infections) and altogether from the epidemiological analyses. The reason for that was that we had dates only for the individual’s first hospital contact for a given infection category; if the mother subsequently received another infection diagnosis from the same category, we would not have the date of contact for it. Therefore, in cases in which an infection occurred before the pregnancy (i.e. earlier than 9 months before the date of birth of the child), we would not be able to rule out that the mother had an infection from the same category during or after the pregnancy. Maternal infections were cross-referenced with iPSYCH individuals using a link between children and mothers from the Danish Medical Birth Register. During this process we discovered that 23 individuals had different IDs for their mothers between the Danish Medical Birth Register and the Danish Civil Registration System in 2016. This could be the result of legal adoptions. Given the focus on pregnancy-related infections in this study, we chose to use the mothers’ IDs from the Danish Medical Birth Register (of note, our infections dataset had some infection diagnoses for the birth mothers of those 23 children, so we know they were included). Lastly, there were 3 pairs of iPSYCH individuals who had the same mother (within each pair) in the Danish Medical Birth Register. Given that first and second degree relatives (based on genetic data) should have been removed during QC, we examined relatedness in the QCed sample using the *–genome* function in PLINK [32] using a pruned set of markers from the QCed pre-imputation dataset (see next section). We found that very few pairs (*N* = 15, out of 65,534 people) had a Pi_hat >0.185, the standard threshold for GWAS [33] (maximum Pi_hat = 0.2672, which was for one of the three aforementioned pairs, who may be half-siblings). It should be noted that the original QC used a different method for determining relatedness (using the KING [34] software), and it was done on the larger non-QCed sample and possibly with a different set of markers. Given the very small number of individuals that this may affect, we chose not to change the QC protocol for this study with regards to these samples. In our previous studies [7, 8, 31, 35], our analyses were blind to the vital status of the individuals, but we now know that by July 2013 a small number of individuals from the QCed sample died, emigrated from Denmark, or otherwise lost contact with the authorities (0.43%, 1.02%, and 0.02% of the sample, respectively). While we confirmed that censoring the age for these people did not have any substantial effect on the highlighted associations in our previous studies, here we chose to censor the age covariates for these people based on the date of death, emigration or loss of contact with the authorities, respectively (i.e. we set their age to what it was when their status changed, whereas, for other individuals, the age at the end of 2013 was used).

### Quality control for genetic markers

Samples were genotyped on the Illumina PsychChip. There were several rounds of QC. Our first dataset had 78,050 samples genotyped in 23 of the original 25 waves. QC steps for this dataset are described elsewhere [3]. This dataset was QCed further, and a full description of the procedure of the sample and marker QC is provided in the first study which utilized it [6] (but note that that study had a minor allele frequency threshold (for dosage data) different from the one below). Sample QC was summarized in the previous section; marker QC steps are described briefly here: before the imputation, markers with rare alleles and non-autosomal markers were removed (for more information about the pre-imputation QC, see one of the first studies which describe the full QC [6, 36]). Genotypes were phased with SHAPEIT3 [37] and imputed with IMPUTE2 [38]. Following the imputation, the following exclusion thresholds for markers were employed: an INFO score (calculated with QCTOOL) < 0.2; a minor allele frequency (MAF) < 0.001; best-guess genotypes missing in >10% of subjects, where the missingness in imputed genotypes was determined by treating individual genotypes with probability < 0.9 as missing for the purpose of this check; Hardy-Weinberg equilibrium *P* < 1 × 10^−6^, or a genome-wide significant association with the genotyping wave or with with the imputation batch itself (in controls in a homogeneous European subset of the sample). Markers with differential missingness across psychiatric cases and controls (*P* < 1 × 10^−6^) were also removed. The dataset used in the analyses in this study included only best-guess genotypes, or hard calls, and only genotypes with probability of 0.9 or above were retained. Markers were retained only if they had an INFO score (following the imputation) of at least 0.8 and MAF of at least 0.01 (in the QCed sample). The final dataset included 7,071,055 markers (the genome build for this dataset was hg19). These QC steps were also employed in our previous analyses of heritability and/or genetic correlation of infections [7, 31].

### Epidemiological analyses

Statistical analyses were performed in R [39] v.3.5.1. For determining the association between a diagnosis of ASD and maternal infection during or shortly after the pregnancy, we performed logistic regressions (confidence intervals for the estimates were obtained using the *confint* function), resulting in a two-sided *p* value from a Wald test for the coefficient’s being different from zero, as implemented in the *glm* function in R. In these analyses, ASD cases had one of the ICD-10 diagnoses as detailed earlier, and ASD controls did not. It was also a requirement for ASD controls to have been included as part of the random population sample, so as to leverage the iPSYCH case-cohort design. With regards to maternal pregnancy-related infections, individuals whose mothers had an infection diagnosis within the time range as described earlier were retained, as were individuals whose mothers did not have any infection diagnosis. Individuals whose mothers had infections only outside the relevant time range for pregnancy-related infections were excluded from these analyses. The regression was performed twice: one time with the ASD outcome regressed on maternal pregnancy-related infections and covariates for age (in years), age squared and sex, and a second time with those covariates as well as a covariate for any having infection diagnosis in the iPSYCH individuals themselves, as our former study found an association between having any infection and having ASD [7]. The sample size for these analyses was 21,817, of whom 7559 had an ASD diagnosis (623 with a history of maternal pregnancy-related infections; 6936 without a history of maternal infections), and 14,258 did not have an ASD diagnosis and were included in the random population sample (825 with a history of maternal pregnancy-related infections; 13,433 without a history of maternal infections). For comparison, we also tested for association between overall infection (in the iPSYCH individuals) and any psychiatric diagnosis in iPSYCH (ICD-10 codes F00-F99 and/or ICD-8 codes 290–315) using logistic regression of the psychiatric disorder on the infection with covariates for age, age squared and sex in the random population sample, as well as a similar regression but with ASD as the outcome. We used the random population sample in the latter two regression analyses, as it allowed for the estimation of population-based estimates, and the phenotypes in question had large enough case sample sizes in the random sample. Lastly, we performed an omnibus test for an association between the ASD subtype (as per the five ICD-10 codes used to define ASD cases) and maternal pregnancy-related infections. To do that, we used ASD cases with a history of maternal pregnancy-related infections, as per the above, and ASD cases without a history of maternal infections, and performed a χ^2^ test with the *chisq.test* function in R using the counts of cases in each subtype in those two groups. We removed individuals who had more than one ASD diagnosis from this test (total number of ASD cases in this test: 6333).

### Genetic analyses

Our genetic analyses included GWAS, heritability estimates and genetic correlation analyses. The GWASs consisted of logistic regressions performed with the *–logistic* test in PLINK v.1.90b6.18. Three GWASs were performed, using the following case and control groups: case group 1 consisted of ASD cases whose mothers had an infection during or shortly after the pregnancy (within the time range) (*N* = 623); case group 2 consisted of ASD cases whose mothers did not have any infection diagnosis in our dataset (*N* = 6936); ASD controls were defined as both not having an ASD diagnosis and as having been included in the random population sample (N = 21,429). The first two GWASs were with case group 1 and controls and case group 2 and controls, respectively. The third GWAS was a case-case GWAS with case group 1 defined as cases and case group 2 defined as controls. All GWASs were run with covariates for age (in years), age squared, sex and the first 10 PCs. LDSC [40, 41] was used to estimate heritabilities and genetic correlations, as it is robust to sample overlap. LD score files were generated using the QC-passing random population sample with a 1 cM window and the genetic map from 1000 Genomes phase 3, as described previously [7]. The Manhattan plot and the QQ plot were generated with the “qqman” R scripts by Stephen Turner and Daniel Capurso (with the (major update) version from April 19, 2011 for the former plot and the version from June 10, 2013 for the latter, available from: https://github.com/stephenturner/qqman/blob/v0.0.0/qqman.r). The summary statistics (PLINK output) from each GWAS were processed with the *munge_sumstats.py* script with the default parameters (after adding A2 from the PLINK bim file and using the NMISS column as the N for LDSC, i.e., non-missing genotypes for cases+controls, as the LDSC tutorial indicated N should be the total number of individuals in the GWAS: https://github.com/Nealelab/ldsc/wiki/Heritability-and-Genetic-Correlation, 9th revision), and LDSC (*ldsc.py*) v.1.0.1 was used (with the default parameters) in the estimation of the heritabilities and genetic correlations. In the iPSYCH datasets, 5,464,859 markers (single-nucleotide polymorphisms (SNPs) that were not strand-ambiguous) remained after the processing with *munge_sumstats.py*.

We tested the heritability (*h*^2^) as being different from 0 using a Wald test and the χ^2^ distribution with 1 degree of freedom (d.f.) in the following way: *χ*^2^ = (*h*^2^/SE)^2^, where *h*^2^ is the heritability on the observed scale from LDSC, and SE is the standard error for the heritability, and *p* values were calculated with 0.5*pchisq(χ^2^, *df* = *1, lower.tail* = *F)* in R (we multiply by 0.5 since *h*^2^ should be non-negative). For the genetic correlation (*r*_g_), we tested it as being different from 0 using a Wald test as well: *χ*^2^ = (*r*_g_/SE)^2^, where *r*_g_ is the genetic correlation from LDSC, and SE is the standard error for the genetic correlation. In some cases, we tested it as being different from 1 with *χ*^2^ = ((*r*_g_−1)/SE)^2^. *P* values were calculated in R using *pchisq(χ*^2^, *df* = *1, lower.tail* = *F)*. Unless otherwise stated, the tested (alternative) hypothesis was that *r*_g_ was different from 0.

To get another perspective on the genetic links between infections and psychiatric disorders in general and ASD in particular, we also performed genetic correlation analyses between having any of the infection categories (susceptibility to overall infection) and either (overall) ASD or any psychiatric diagnosis in iPSYCH (ICD-10 codes F00-F99 and/or ICD-8 codes 290–315). The latter was performed in an earlier study of ours [7], but we repeated this analysis using the exact same processing steps with LDSC and covariates as in the other analyses in the current study. Note that the GWAS for overall infection in these analyses did not have a covariate for psychiatric diagnosis. Lastly, heritabilities (but not genetic correlations, due to control sample overlaps) were also calculated with GCTA [42]. The genetic relationship matrix was calculated for each autosomal chromosome separately with *–make-grm* and merged with *–mgrm* with GCTA v1.91.1 beta as previously described [31]. Heritability for the ASD phenotypes (and for case-case comparisons) was calculated with *–reml* in GCTA v1.93.2 beta with covariates for age, age squared, sex and the first 10 PCs. The likelihood ratio test statistic from the GCTA output was used to derive *p* values using the χ^2^ distribution with d.f. = 1 as described above for the LDSC *h*^2^ estimates.

### Replication sample and internal controls

To replicate the results of our primary genetic correlation analysis for the two main ASD phenotypes, we used summary statistics from the most recent PGC GWAS for ASD [3], but rather than using summary statistics from the analysis described in the paper, we used summary statistics from a meta-analysis which excluded the iPSYCH samples (without iPSYCH, *N* = 5305). As specified in the paper, these samples were QCed and were of European ancestry. Marker names were changed based on chromosome number and position to correspond to the marker names in the iPSYCH dataset (both datasets were in build hg19), and only the markers which were also found in the iPSYCH dataset based on chromosome number and position were kept. Additionally, we retained only markers which had an INFO score ≥ 0.8 and MAF (in both case and control columns) ≥ 0.01, to conform to our own QC protocol. A small number of markers with duplicate positions were found and subsequently removed. The resulting subset of the summary statistics was processed with *munge_sumstats.py* as described earlier (5,389,411 markers remained). We then performed genetic correlation analyses between our phenotypes and the ASD phenotype from the PGC study. It should be noted that the non-iPSYCH PGC ASD sample used cases from family trios and matched pseudocontrols (created with the non-transmitted alleles from the parents). Using either the *N*_eff_ sample size (from the summary statistics, which was also the number of individuals in the sample) or twice that number (as there were as many pseudocontrols as there were cases) as the N for LDSC resulted in the same genetic correlations with our phenotypes and the same SEs. Similarly, providing the population and sample prevalences to LDSC (for the PGC phenotype, using a population prevalence of 0.01 [43] and sample prevalence of 0.5 and *N* for cases+pseudocontrols, and, for the iPSYCH phenotypes, using population prevalences estimated from the QCed random population sample (although they are not accurate lifetime prevalences due to the age limitations of the sample) and the sample prevalences from the GWASs) when estimating the genetic correlations resulted in the same estimates and SEs as when not specifying these. These changes affect the observed scale heritabilities, genetic covariances, and the transformation to the liability scale, but we did also verify that the genetic correlations in the LDSC output did not change, as per the above (theoretically, there is no distinction between observed and liability scale genetic correlation for case/control traits [41]).

For the purpose of comparison with our two ASD phenotypes, we also employed two internal controls and performed two additional case–control GWASs: one for ASD within the full iPSYCH ASD sample (i.e., irrespective of maternal infections, with 12,331 cases), and another with ASD cases from iPSYCH whose mothers had infections only more than 2 months after the birth of the iPSYCH individual (1861 cases), and estimated the genetic correlations of these phenotypes with the PGC ASD phenotype. The latter group (ASD cases with a history of maternal infections occurring only more than 2 months after their birth) was also used in a second case-case GWAS design as cases, together with ASD cases with no history of maternal infections as controls. Lastly, in order to assess the potential bias in the genetic correlations from the difference in sample size between the ASD group with a history of maternal pregnancy-related infections (*N* = 623) and the ASD group without maternal history of infections (*N* = 6936), we performed 10 GWASs for the latter group, randomly selecting 623 cases for each GWAS from the 6936 cases for that phenotype. We then performed genetic correlation analyses with the PGC replication sample and calculated the average *r*_g_.

## Results

Of our QCed sample of 65,534 individuals from the iPSYCH study, 12,331 had a diagnosis of ASD, and 21,429 individuals were controls for ASD from the random population sample. We identified 623 ASD cases whose mothers had pregnancy-related infections. There were 6936 ASD cases with no history of maternal infection. These were the two main case phenotypes in this study. Additionally, there were 1861 ASD cases with maternal infections which occurred only more than 2 months following their birth, and these were included in an internal control analysis (Methods). ASD cases with a history of maternal infections occurring only outside the above ranges were not used (except for in analyses using all ASD cases irrespective of maternal infections).

### Epidemiological analyses

When regressing the ASD diagnosis on the indication of maternal pregnancy-related infections with covariates for sex, age, and age squared, maternal pregnancy-related infections were associated with ASD (in the offspring): OR = 1.343 (95% CI: 1.197–1.507, *P* = 5.15 × 10^−7^). When further adding to the model a covariate for any infection diagnosed in the offspring, the association is slightly reduced at OR = 1.284 (95% CI: 1.143–1.441, *P* = 2.42 × 10^−5^). In general, the association between having any infection and any psychiatric diagnosis (in iPSYCH), both diagnosed in the offspring generation, was stronger (OR = 1.733, 95% CI: 1.584–1.896, *P* = 4.33 × 10^−33^), and the same was observed for ASD (OR = 1.556, 95% CI: 1.223–1.977, *P* = 0.0003), in line with a previous study [7]. We also performed an omnibus test for an association between ASD diagnostic subtypes (as per the ICD-10 codes which make up our ASD phenotype) and maternal pregnancy-related infections. The test did not identify a significant association (*χ*^2^ = 9.285, d.f. = 4, *P* = 0.0544).

### Genetic correlation analyses

In the genetic analyses, both ASD phenotypes (ASD with a history of maternal pregnancy-related infections, and ASD with no history of maternal infections) showed heritabilities significantly different from zero using LDSC: observed scale *h*^2^ = 0.0409 (SE = 0.0113, *P* = 0.0001) and *h*^2^ = 0.0869 (SE = 0.0117, *P* = 5.54 × 10^−14^), respectively. The genetic correlation between these two ASD phenotypes was *r*_g_ = 0.3811 (SE = 0.1299, *P* = 0.0033, when testing against a null of *r*_g_ = 0; *P* = 1.89 × 10^−6^ when testing against a null of *r*_g_ = 1). As an internal genetic control, we estimated the genetic correlation between ASD with no history of maternal infections and ASD with a history of maternal infections occurring only more than 2 months following childbirth; we obtained *r*_g_ = 1.1199 (SE = 0.2565). When testing against a null of *r*g = 1, we obtained *P* = 0.6402. Note that LD score regression is not a bounded estimator, and so an *r*_g_ outside [−1,1] can be obtained, possibly due to sampling variation. However, if the estimate is not extreme (by default LDSC issues a warning only if the estimate is above 1.25 or below −1.25), it can still be interpretable. Therefore, we interpret this result to mean that there is no significant evidence to the effect that the two ASD phenotypes in question differ genetically. Similar to our previous report, the genetic correlation between (susceptibility to) overall infection and psychiatric diagnosis was high: *r*_g_ = 0.4061 (SE = 0.1049, *P* = 0.0001). However, when examining ASD specifically, there was no significant genetic correlation with overall infection: *r*_g_ = −0.0126 (SE = 0.1061, *P* = 0.9055). The former result in particular should be evaluated with caution, since it was measured between a primary phenotype and a secondary phenotype that show comorbidity. The heritability estimates from GCTA showed similar trends: for ASD with a history of maternal pregnancy-related infections and ASD with no history of maternal infections, these were *h*^2^ = 0.0454 (SE = 0.0140, *P* = 0.0005) and *h*^2^ = 0.1290 (SE = 0.0120, *P* = 3.17 × 10^−32^), respectively.

The summary statistics from the PGC replication sample were for a general ASD outcome (based on the low frequency of confirmed ASD cases with a history of maternal pregnancy-related infections in our total ASD sample (which is indicative of all ASD cases identified in Denmark), we assume that most ASD cases in the PGC did not have a history of maternal pregnancy-related infections). The genetic correlation between ASD with a history of maternal pregnancy-related infections and the PGC ASD phenotype was *r*_g_ = 0.3997 (SE = 0.1552, P = 0.0100, not surviving Bonferroni correction for the total number of *r*_g_s estimated, *N* = 6, but surviving a correction for the number of *r*_g_s estimated with the replication sample, *N* = 4). The genetic correlation between the ASD phenotype with no history of maternal infections and the PGC ASD phenotype was *r*_g_ = 0.6735 (SE = 0.0944, *P* = 9.71 × 10^−13^). When testing the genetic correlation between overall ASD in the iPSYCH sample (irrespective of maternal infections) and the PGC ASD phenotype, the correlation was *r*_g_ = 0.6344 (SE = 0.0830, *P* = 2.12 × 10^−14^). When using ASD cases whose mothers had infections occurring only more than 2 months after childbirth, the genetic correlation with the PGC ASD phenotype was *r*_g_ = 0.6293 (SE = 0.2134, *P* = 0.0032). This result indicates that the infection’s temporal relation to the pregnancy may have reduced the genetic correlation to < 0.6, but it should be noted that, while the PGC sample allowed us to check genetic correlation trends with our phenotypes, it was not a full replication sample, in the sense that we did not have raw genotype data and maternal infection data for that sample, so we could not do exactly the same analyses as in iPSYCH. Table 1 summarizes the main genetic correlation analyses. Lastly, when randomly selecting 623 ASD cases without a history of maternal infections ten times and performing GWASs and genetic correlation analyses with the PGC ASD phenotype, the average *r*_g_ across all instances with *P* ≤ 0.05 was 0.6742 with a standard deviation (SD) of 0.1184. Across all *r*_g_s obtained in this test, irrespective of the *p* value, the average *r*_g_ was 0.6561 (SD = 0.1747). One *r*_g_ was excluded in this analysis because one of the *h*^2^s for it was out of bounds.

**Table 1:** LDSC genetic correlation analyses between the various phenotypes, including internal controls and replication with PGC.

Phenotype 1 (numbers of case;controls)	Phenotype 2 (numbers of case;controls)	*r*_g_	SE	*P*
iPSYCH ASD with a history of maternal pregnancy-related infections (623;21,429)	iPSYCH ASD with no history of maternal infections (6936;21,429)	0.3811	0.1299	0.0033
iPSYCH ASD with a history of maternal pregnancy-related infections (623;21,429)	PGC ASD phenotype^a^ (replication) (5305;5305)	0.3997	0.1552	0.0100
iPSYCH ASD with no history of maternal infections (6936;21,429)	PGC ASD phenotype (replication) (5305;5305)	0.6735	0.0944	9.71 × 10^−13^
iPSYCH ASD irrespective of maternal infections (12,331;21,429)	PGC ASD phenotype (replication) (5305;5305)	0.6344	0.0830	2.12 × 10^−14^
iPSYCH ASD with maternal infections occurring only more than 2 months after childbirth (1861;21,429)	iPSYCH ASD with no history of maternal infections (6936;21,429)	1.1199	0.2565	1.26 × 10^−5^
iPSYCH ASD with maternal infections occurring only more than 2 months after childbirth (1861;21,429)	PGC ASD phenotype (replication) (5305;5305)	0.6293	0.2134	0.0032

The *p* values are for tests for a difference from an *r*_g_ of zero.

*SE* standard error; *r*_*g*_ genetic correlation estimate; *P* *p* value.

^a^The PGC study used cases and pseudocontrols.

### Case-case GWAS for the main ASD phenotypes

**Fig. 2:**
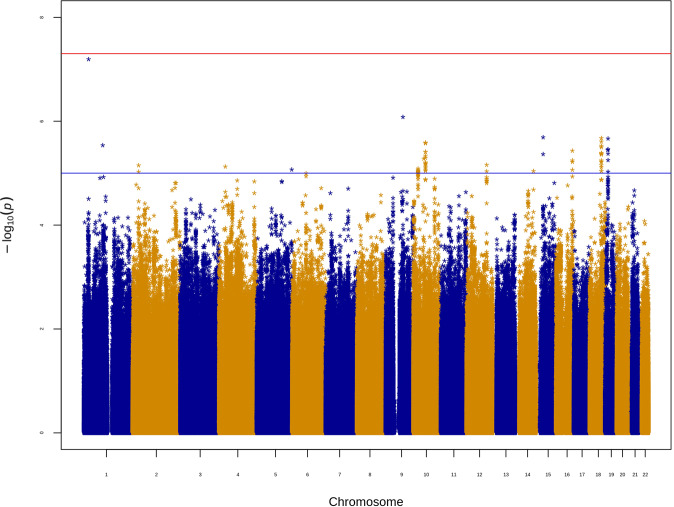
Manhattan plot for the case-case GWAS between ASD with a history of maternal pregnancy-related infections and ASD with no history of maternal infections. The blue line represents the threshold for suggestive association (*P* = 1 × 10^−5^), and the red line represents the threshold for genome-wide significance (*P* = 5 × 10^−8^).

We performed a case-case GWAS with the two main ASD case groups and estimated the “heritability” directly from that with LDSC, obtaining *h*^2^_cc_ = 0.1059 (SE = 0.0313, *P* = 0.0004). It should be noted that the estimate from this analysis is not to be interpreted as a heritability estimate for a disease in the traditional sense, i.e., as when looking at a binary trait with case–control data; rather, we used this analysis to estimate the genetic differences between the two ASD groups directly; if cases of one phenotype are more similar (genetically) within themselves, and cases of the other phenotype are also more similar (genetically) within themselves, then this would result in increased genetic variance across the groups and hence a non-zero heritability estimate [44]. Barring any QC-related artifacts, which should not be present in our analyses, as individuals from both case groups were ascertained on the basis of ASD status and underwent the same collection, genotyping and QC procedures, this would support the possibility of different ASD etiological subtypes, from the genetic perspective. We therefore refer to this estimate as case-case heritability (*h*^2^_cc_) to distinguish it from the case-control heritability estimates elsewhere in the paper. The top association in the GWAS was with marker rs115840688, which nearly achieved genome-wide significance (OR = 2.159 effect allele: A, other allele: T, *P* = 6.39 × 10^−8^). The second most significant association was with rs116870656 (OR = 2.301 effect allele: C, other allele: T, *P* = 8.31 × 10^−7^). Supplementary Table S2 includes all suggestive associations (*P* ≤ 1 × 10^−5^) from this GWAS. Figure 2 shows the Manhattan plot for the case-case GWAS, and Supplementary Fig. S1 shows the corresponding QQ plot. For comparison with this analysis, we also performed a second case-case GWAS with the following two groups: ASD cases with a history of maternal infections occurring only more than 2 months after childbirth (defined as cases) and ASD cases with no history of maternal infections (defined as controls). In contrast to the previous result, there was no indication of significant overall genetic differences between the groups (*h*^2^_cc_ = −0.0203, SE = 0.0323, P = 0.2648). Note that a negative *h*^2^ usually means that the true heritability is close to zero. According to the LDSC website, if this happens and the mean χ^2^ is not above ~1.02 (which it is not, in our case), it means there is very little polygenic signal, as would be expected if the two groups did not differ genetically. The GCTA estimates showed similar trends: *h*^2^_cc_ = 0.0806 (SE = 0.0392, *P* = 0.0180) and *h*^2^_cc_ = 0.0239 (SE = 0.0336, *P* = 0.2444) for the first and second case-case GWAS phenotypes, respectively.

## Discussion

In this study we examined genetic correlations between potential ASD etiological subtypes stratified on the basis of a history of maternal pregnancy-related infections. We replicated the previously reported epidemiological observation of increased risk of ASD in the child conferred by maternal pregnancy-related infections. This effect was observed even when adjusting the model for infections diagnosed in the child. In addition to confirming the presence of this effect in our sample, this also supported the adequacy of the time range selected for the genetic analyses. When comparing this result to the direct association between infection in the child and ASD, the OR is higher for the latter. Thus, the effect of having an infection on the odds of having ASD is higher than the effect of the mother’s having had a pregnancy-related infection on the odds of having ASD, but the latter remains significant even when adjusting for the infection in the child (also, in the case of the association between the mother’s pregnancy-related infection and ASD risk, there is a clear temporal relation, whereas the association between the child’s infection and ASD diagnosis as modeled in this study does not entail such a relation). Interestingly, while the epidemiological association exists on both of these generational levels, we observed no significant genetic correlation between ASD and overall susceptibility to infection.

In the genetic analyses, we observed that stratifying individuals with ASD based on exposure to maternal pregnancy-related infections highlighted genetic differences between them. First, both the heritability of ASD with a history of maternal pregnancy-related infections and that of ASD with no history of maternal infections were significantly different from zero, suggesting that genetic variants predispose individuals to their respective ASD phenotypes in both groups. That said, as the heritability estimates are on the observed scale (there are currently no reported population lifetime prevalences for these phenotypes), we do not interpret these results in a direct quantitative comparison between the two phenotypes. With regards to the former phenotype, this suggests that, while environmental factors (arguably the maternal infection itself) may play a role in the development of ASD in children whose mothers had a pregnancy-related infection, genetic predisposition still exists. Moreover, the positive and significant genetic correlation between the two ASD phenotypes suggests that some of this genetic predisposition is related to genetic risk of ASD in general, as the estimate is significantly different from zero. However, our results also indicate that their genetic correlation is significantly different from one, suggesting that they may have different, albeit overlapping etiologies, from the genetic perspective. These observations could result from several scenarios or a combination thereof; for example, there could be genetic differences between both ASD case groups on the one hand, and ASD controls on the other hand, and there could be phenotype-specific genetic risk factors, as illustrated by the *h*^2^_cc_ estimate derived from the first case-case GWAS. In this respect, one needs to consider the interpretation of this result; the difference between the case groups based on which we stratified the sample was the presence or absence of maternal infections (and, if present, during or shortly after the pregnancy). Clearly, however, we are not estimating the heritability of this “trait” (the notion that this is the only difference would also presuppose that the ASD outcomes in both case groups are identical, which, we hypothesize, is not the case). Rather, we hypothesize that there are different routes to ASD, which may differ genetically. With this in mind, the second case-case GWAS (comparing ASD with no history of maternal infections and ASD and a history of maternal infections occurring only more than 2 months following childbirth), which did not result in a non-zero heritability, adds further support to this hypothesis. Thus, genetic risk factors unique to ASD with a history of maternal pregnancy-related infections could also include genetic modulation of immune-related risk. Moreover, the lack of significant genetic correlation between ASD and overall susceptibility to infection (when using the iPSYCH individuals themselves for both traits) implies that it is not the case that pleiotropy between these last two traits is driving the main observations (through the inheritance of common ASD-infection susceptibility risk variants from the mother). It is worth mentioning that a similar approach was developed to study single-variant differences between genetically correlated diseases; this approach uses summary statistics from case-control GWASs for individual diseases, but the results were concordant with using individual-level data from a direct case-case GWAS [45]. Similarly, a recent study examining potential genetically different subtypes of major depression utilized a strategy whereby genetic correlations between the subtypes (with overlapping controls, who, in most analyses, were defined as simply not having the main outcome of major depression) were estimated; some of these subtypes included diagnostic criteria which, albeit biological, could also be thought of as external e.g., depression related to childbirth, and, indeed, in many cases the genetic correlation was significantly different from both zero and one [46].

One way to interpret these findings is in the context of polygenicity and liability [47, 48]: it could be that the ASD risk variants carried by individuals with the phenotype of ASD with a history of maternal pregnancy-related infections are not sufficient to push these individuals over the liability threshold for ASD, but, together with other risk factors (namely, one or a both of: the direct consequences of maternal pregnancy-related infections, and genetic variants in the child that alter the response to these consequences) they do lead to ASD. It could also be the case that it is only further environmental risk factors that lead to ASD in these individuals; although the results of our first case-case GWAS may suggest relevant genetic differences between the ASD phenotypes. While this case-case GWAS did not detect genome-wide significant associations, the top associations are related to infection or immunity: the top marker in the GWAS for ASD with a history of maternal pregnancy-related infections (vs. ASD controls) was rs115840688, which achieved genome-wide significance in that GWAS (OR = 2.166 effect allele: A, other allele: T, *P* = 1.33 × 10^−8^). The same marker almost achieved genome-wide significance and was the top marker in the first case-case GWAS (OR = 2.159 effect allele: A,other allele: T, *P* = 6.39 × 10^−8^), illustrating the fact that, for some variants, cases of this ASD phenotype are almost as different from ASD controls as they are different from ASD cases with no history of maternal infections. On the Ensembl genome browser, this variant falls within a transcript of the *SRRM1* gene (ENST00000478890.1, hg19), which has been implicated in Parkinson dementia, a disease associated with neuroinflammation [49], although this transcript is listed as not containing an open reading frame. A recent study showed that its protein product co-purified with endogenous positive coactivator 4 (PC4, encoded by the *SUB1* gene) from B cells, which, in turn, is important for B cell differentiation [50]. The second highest ranking marker was rs116870656 (OR = 2.301 effect allele: C, other allele: T, *P* = 8.31 × 10^−7^). This marker falls within *NTRK2*, a gene whose mouse ortholog has been implicated in splenomegaly in *L. donovani* infection [51].

### Limitations of our study

The main limitation of our study is that the number of ASD cases with a history of maternal pregnancy-related infections was small. This can affect downstream analyses such as genetic correlation analysis and GWAS. In order to tackle the issue, we employed three strategies: (i) we used an internal control (ASD cases with a maternal history of infection occurring only more than 2 months following childbirth) to confirm that the timing of the infection in the mother is important; (ii) we used the PGC ASD sample as replication sample for the genetic correlations, under the assumption that most of its ASD cases do not have a history of maternal pregnancy-related infections (this is what we see in our sample, which includes almost all ASD cases in the country (born in 1981–2005) by design); (iii) we used a repeated random sampling procedure with the larger of the two main case groups, selecting at random the same number of cases as in the smaller case group, to assess the effect of the smaller sample size on the genetic correlation. The small sample size, however, could also impact the power of the case-case GWAS, for which we did not have a suitable replication sample. Therefore, the results of this GWAS, directly assessing the genetic differences between the case groups, should be considered at most suggestive.

Some caution regarding the interpretation of the results of the genetic analyses is also important. In these analyses, our goal was to assess the genetic differences and similarities between two potential ASD subtypes. Therefore, unlike in the epidemiological analyses, which tested the direct effect of maternal pregnancy-related infections on ASD risk, we were agnostic about maternal infection in ASD controls, and the control subsets in the iPSYCH ASD genetic analyses overlapped, an approach which has been used to study subtypes of major depression [46]. Any genetic differences, therefore, apart from sampling variation and case sample size issues (which we tried to tackle as per the above), were expected to result from differences between the ASD case groups. The trends were then confirmed with the case-case GWASs, and these two approaches should be viewed as complementary. The advantage of the former is that we could use summary statistics from an external study (the PGC ASD study) to try to replicate our results, and the advantage of the latter is that it compares case groups directly and allows the identification of specific genetic variants that differ between them. Given that the genetic overlap between overall ASD in iPSYCH and the PGC ASD phenotype was not 1, it makes the quantitative interpretation of the combined results difficult. This is also the case in the original study from which the summary statistics are derived (note again that we used summary statistics from a meta-analysis without the iPSYCH samples); the genetic correlation between the iPSYCH ASD phenotype and the PGC ASD phenotype was not 1, although it was higher than in this study at *r*_g_ = 0.779 (this is likely due to the different sample QC employed here, different imputation pipelines and different marker datasets) [3]. This suggests that there could be some inherent difference between iPSYCH and PGC in terms of the ascertainment of ASD cases. Therefore, we view our results as indicative of genetic differences between ASD subtypes based on a history of maternal pregnancy-related infections, but an accurate quantification of those differences may require a study designed specifically for this purpose. Lastly, it should be mentioned that the ASD phenotype used in this study consisted of five ICD codes. It is possible that we are detecting genetic differences across these ASD subtypes, if they are associated with maternal pregnancy-related infections to different degrees. Our omnibus test did not detect a significant association between the two ASD phenotypes used in the primary analyses and the ICD-based ASD subtypes at *α* = 0.05, but the *p* value was not very much greater than that. Thus, a study with larger sample sizes of clinical ASD subtypes is needed to investigate this further.

In conclusion, the results of our study suggest that there may be genetically different etiological subtypes of ASD based on a history of maternal pregnancy-related infections, which share some genetic predisposing factors, but may differ in others, some of which could be related to immune-related genes. In other words, there could be different routes to ASD in the child based on their exposure to infections during pregnancy or in early development. Larger studies, where both maternal infection data and ASD phenotype data, and potentially more immune-related data, are available would be needed in order to identify specific underlying genetic factors that may distinguish the main two ASD phenotypes investigated in this study. While it is possible that some of the estimates reported here were influenced by the small number of cases of ASD with a history of maternal pregnancy-related infections, our results nonetheless suggest that future studies, in particular genetic studies, may benefit from taking into account this potential difference.

## Supplementary information

Supplementary Figure S1

Supplementary Table S1

Supplementary Table S2

## Data Availability

iPSYCH data are stored in a national HPC facility in Denmark. The iPSYCH initiative is committed to providing access to these data to the scientific community, in accordance with Danish law. Researchers may be granted access upon request to the iPSYCH management. Summary statistics are available from the corresponding author upon reasonable request.
