# Supplementary figures and images for: Maternal pregnancy-related infections and autism spectrum disorder—the genetic perspective
Source: Transl Psychiatry. 2022 Aug 16;12:334. doi: 10.1038/s41398-022-02068-9 (PMC9381559; doi:10.1038/s41398-022-02068-9)

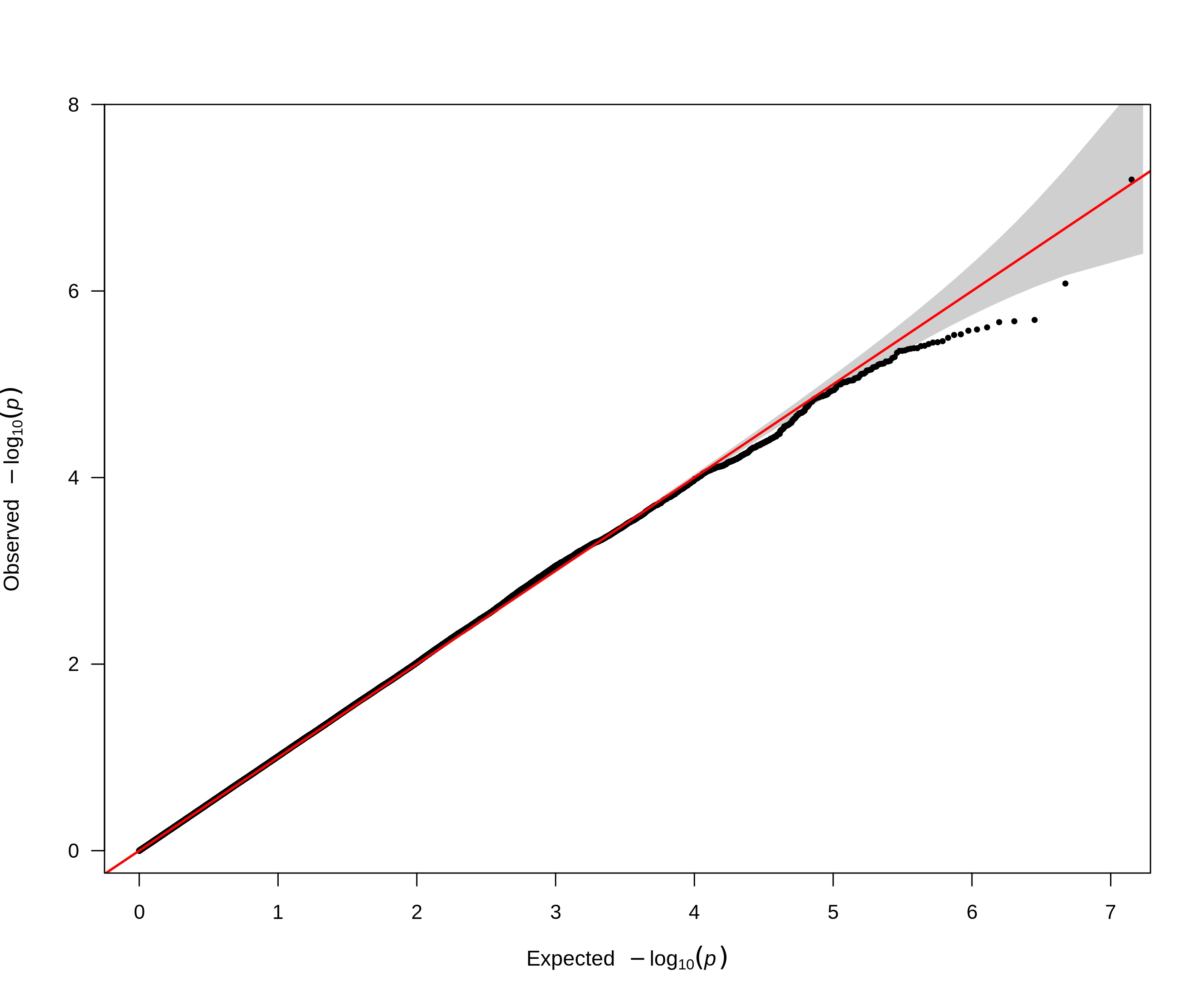

Supplement: Supplementary file 1 — Supplementary Figure S1 [file 41398_2022_2068_MOESM1_ESM.jpg]
