# Supplementary material for: Maternal pregnancy-related infections and autism spectrum disorder—the genetic perspective
Source: Transl Psychiatry. 2022 Aug 16;12:334. doi: 10.1038/s41398-022-02068-9 (PMC9381559; doi:10.1038/s41398-022-02068-9)
Supplement: Supplementary file 2 — Supplementary Table S1 [file 41398_2022_2068_MOESM2_ESM.pdf]

Supplementary Table S1: ICD-8 and ICD-10 codes for site and type of infection		
Infection category	ICD-8 Codes	ICD-10 Codes
**Site of infection**		
Sepsis infections	038	A40-A41
Hepatitis infections	070	B15-B19, K770A, K770B
Gastrointestinal infections	000-009, 540	A00-A09, K35
Skin infection	035, 050-057, 110-111, 680-686	A46, B00-B09, L00-L08
Respiratory infections	460-486	J00-J18, J22, J36
Urological infections	580, 590, 595	N00, N05, N129, N300, N370, N390
Genital infection	604, 612, 620, 622	N45, N512, N70, N700, N709, N760, N762, N764, N770, N771, N518B
Pregnancy-related infections	630, 635, 670	O23, O264, O85-O86, O98
Otitis media infections	381-382	H65-H67
Central nervous system infections	013, 02701, 03609, 040-043, 04509, 04519, 04599, 046, 05201, 05302, 05403, 05501, 05601, 06209, 06219, 06229, 06239, 06249, 06299, 06309, 06319, 06329, 06399, 06499, 06599, 07199, 07202, 07501, 07929, 09049, 09490, 0949, 320, 322-324, 474	A066, A022C, A17, A229C, A321, A390, A504, A514B, A521, A521A, A521B, A548A, A548D, A80-A89, B003, B004, B010, B011, B020, B021, B050, B051, B060, B261, B262, B375, B451, B582, B602, E236A, G00-G07
HIV/AIDS	07983	B20-B24
**Type of infection**		
Bacterial infections	000, 00009, 00019, 00099, 00199, 002-005, 008, 01099, 011-018, 020-023, 025-027, 030-039, 07399, 07699, 07984, 08899, 08900, 08099, 081-083, 09009, 09049, 09059, 091-098, 100-104, 32009, 32019, 32080, 322, 380-382, 390-392, 420-421, 461-463, 46403, 481-483, 50199, 50800, 50801, 50802, 50803, 510, 513, 52259, 52649, 52722, 52838, 52839, 52903, 54001, 566-567, 57703, 590, 59500, 59501, 59700, 59703, 59900, 59906, 601, 604, 60739, 61100, 61101, 612, 614, 62090, 62099, 622, 62949, 630, 63109, 63110, 63111, 63119, 63129, 63139, 635, 670, 67801, 680, 68108, 68109, 682, 68399, 68408, 68409, 68501, 68600, 68608, 710, 7200, 7201, 7202, 72031, 73299	A022C, A03-A05, A15-A28, A30-A58, A65-A79, B95-B96, B088D, D733, E060A, E236A, E321, I301A, I301B, I301C, I301D, I320, I410, I430, I520, J01, J020, J030, J13-J15, J160, J170, J851, J86, J860, J869, K040A, K046A, K052A, K112A, K130A, K140A, K209A, K113, K122, K351, K61, K650N, K67, K630, K930, L00-L04, L08, M00, M010-M013, M015B, M014, M463, M490-M492, M680, M725A, N10, N12, N136, N151, N200I, N201I, N300, N309, N340, N341, N390, N410, N412, N431, N450, N459, N510A, N510C, N511, N70, N71, N72, N73, N74, N740, N741, N742, N743, N744, N764, N980, O23, O753, O85-O86, O980, O981, O982, T793, T802, T814, T874, T880
Viral infections	00880, 00889, 00890, 040-046, 050-057, 060-065, 067-068, 070-072, 074-075, 46099, 464, 46599, 47099, 471-474, 48099	A08, A60, A630, A80-A89, A90-A99, B00, B060, B01-B09, B15-B26, B260, B261, B262, B263, B268, B269, B27, B270, B271, B278, B279, B33, B330, B331, B332, B333, B338, B34, B340, B341, B342, B343, B344, B348, B349, B97, G020, G051, H621A, H621B, H671A, H671B, I301E, I400B, I411A, I411B, J00, J04-J06, J10- J12, J050, J171, J203, J204, J205, J206, J207, J210, K770A, K770B, K871A, K871B, M014, M015, M015A, N518B, N770D, N771B, N771G, N771L
Other infections	006-007, 00899, 00999, 084, 087, 089, 09991, 09992, 09993, 09999, 110-117, 120-131, 13603, 13600, 572, 99859	A06-A07, A085, A09, A59, A63-A64, B35-B60, B64-B83, B87-B89, B99, G02, G040, G049, G052, G079D, H622, I301, I400, I411, I412, I521C, I33, J18, J02-J03, J172-J173, J178, J22, J998B, J998C, K770C, K770D, K770E, K35, K750, L303, M016, M631C, M631D, M631E, M631F, M632A, M651, M711, N370A, N160D, O983, O986-O989, T89

Notes for this table: we have amended this table to reflect the exact codes that were extracted from the register, as we discovered that the previous versions (1, 2) had some minor errors/omissions. This involved 4 codes that were in the original table but not included in the study (in the viral or bacterial infection categories, namely I398, N11, 078 and 07983, the latter of which was mistakenly written as 079.82), and 4 codes that were included but were not noted in original the table (also in the viral or bacterial infection categories, namely N309, N340, I301E and J050). Regarding the former group, however, the frequency of these codes among individuals born between 1981 and 2005 in the entire Danish population from hospital registry data until 2017 (roughly the same year of birth range and an even later registry access date as in iPSYCH) are 0.000009, 0.00039, 0.000019 and 0.0000098, respectively (the last frequency corrects a typographical error as well (3)). Therefore, we do not expect to have many individuals in iPSYCH who had only these codes and none of the other ones, who were consequently erroneously treated as controls (since the iPSYCH sample was nested in a smaller sample than the one used above and underwent case or random sample selection, as well as quality control, which greatly reduced the final sample). Also, not all codes are, in fact, infection diagnoses per se (I398, N11).

Additionally, we provide a higher resolution (more specific) code for some diagnoses; in most cases this does not reflect any change e.g. where the previous version listed ICD-8 code “062” and this version lists “06209, 06219, 06229, 06239, 06249, 06299”, and only these six codes appear in the Danish ICD-8 under 062. Cases in which ICD-8 had more codes than listed here include 089 and 599, but overall these were very rare, with frequencies of 0.000002 and 0.00004, respectively, for the higher resolution codes that were not included in the study. When a lower resolution code is listed in the table, it can be understood as including all diagnoses under its ICD entry. For example, ICD-8 060 includes 06009, 06019 etc.

1. Nudel R, Appadurai V, Schork AJ, Buil A, Bybjerg-Grauholm J, Borglum AD, et al. A large population-based investigation into the genetics of susceptibility to gastrointestinal infections and the link between gastrointestinal infections and mental illness. Human genetics. 2020;139(5):593-604. Epub 2020/03/11.
2. Nudel R, Wang Y, Appadurai V, Schork AJ, Buil A, Agerbo E, et al. A large-scale genomic investigation of susceptibility to infection and its association with mental disorders in the Danish population. Translational psychiatry. 2019;9(1):283. Epub 2019/11/13.
3. Nudel R, Allesoe RL, Thompson WK, Werge T, Rasmussen S, Benros ME. A large-scale investigation into the role of classical HLA loci in multiple types of severe infections, with a focus on overlaps with autoimmune and mental disorders. Journal of translational medicine. 2021;19(1):230. Epub 2021/06/02.
