# Supplementary material for: Maternal pregnancy-related infections and autism spectrum disorder—the genetic perspective
Source: Transl Psychiatry. 2022 Aug 16;12:334. doi: 10.1038/s41398-022-02068-9 (PMC9381559; doi:10.1038/s41398-022-02068-9)
Supplement: Supplementary file 3 — Supplementary Table S2 [file 41398_2022_2068_MOESM3_ESM.pdf]

Supplementary Table S2: Suggestive associations from the case-case GWAS between ASD with a history of maternal pregnancy-related infections and ASD with no history of maternal infections.

Marker	CHR	BP	A1_effect	A2_other	OR	P
rs115840688:24965155:T:A	1	24965155	A	T	2.159	6.39E-08
rs116870656:87424241:T:C	9	87424241	C	T	2.301	8.31E-07
rs28499090:36981867:G:A	15	36981867	A	G	0.5161	2.04E-06
rs77055220:59309296:T:C	18	59309296	C	T	0.5716	2.11E-06
rs113593840:15128181:AG:A	19	15128181	A	AG	1.545	2.16E-06
rs73965710:59279082:C:T	18	59279082	T	C	0.5759	2.45E-06
rs72815824:61443102:G:A	10	61443102	A	G	1.455	2.59E-06
rs78758931:61442637:TA:T	10	61442637	T	TA	1.454	2.66E-06
rs116019520:96532142:G:T	1	96532142	T	G	2.434	2.91E-06
rs77936581:59313398:A:T	18	59313398	T	A	0.5781	2.96E-06
rs73965711:59279991:C:T	18	59279991	T	C	0.5794	3.18E-06
rs35983811:15133289:C:T	19	15133289	T	C	1.457	3.45E-06
rs143215045:13805880:CTG:C	19	13805880	C	CTG	1.678	3.54E-06
rs34158429:13803626:T:C	19	13803626	C	T	1.677	3.57E-06
rs34803931:83146358:T:G	16	83146358	T	G	0.7543	3.71E-06
rs72812337:61426082:G:A	10	61426082	A	G	1.443	3.86E-06
rs72812320:61401338:A:T	10	61401338	T	A	1.443	3.90E-06
rs76637859:59314388:G:C	18	59314388	C	G	0.5822	4.10E-06
rs9960598:59314289:T:A	18	59314289	A	T	0.5822	4.10E-06
rs73965706:59276522:T:G	18	59276522	G	T	0.5849	4.15E-06
rs71276886:15130781:A:AT	19	15130781	AT	A	1.511	4.22E-06
rs11856353:36971349:T:C	15	36971349	C	T	0.5401	4.33E-06
rs116565907:59315708:G:A	18	59315708	A	G	0.5831	4.38E-06
rs140407434:59316153:T:TC	18	59316153	TC	T	0.5831	4.38E-06
rs12572003:61444695:G:A	10	61444695	A	G	1.48	4.59E-06
rs72812351:61440582:T:G	10	61440582	G	T	1.436	5.10E-06
rs7099535:52441797:T:C	10	52441797	C	T	1.588	5.24E-06
rs71334754:15128105:G:A	19	15128105	A	G	1.521	5.61E-06
rs7196746:83144899:T:A	16	83144899	T	A	0.759	5.70E-06
rs7234372:59314603:T:C	18	59314603	C	T	0.5995	5.70E-06
rs60693457:59261822:G:A	18	59261822	A	G	0.5901	5.97E-06
rs12455735:59316220:C:T	18	59316220	T	C	0.6003	6.03E-06
rs7191965:83144957:A:G	16	83144957	A	G	0.7596	6.06E-06
rs72812334:61425315:A:G	10	61425315	G	A	1.432	6.17E-06
rs117103609:61419989:T:C	10	61419989	C	T	1.431	6.45E-06
rs143636116:61421594:A:C	10	61421594	C	A	1.431	6.52E-06
rs67656598:61388832:A:G	10	61388832	G	A	1.432	6.59E-06
rs17446017:103144758:G:T	12	103144758	T	G	1.748	6.93E-06
rs111900668:59367726:T:C	18	59367726	C	T	0.6126	7.00E-06
rs12328494:30268081:C:T	2	30268081	T	C	1.389	7.08E-06
rs138076534:61424745:CAT:C	10	61424745	C	CAT	1.429	7.13E-06
rs59111564:31787027:G:A	4	31787027	A	G	1.779	7.50E-06
rs16913940:61431755:A:C	10	61431755	C	A	1.427	7.69E-06
rs17068735:59314899:C:T	18	59314899	T	C	0.6052	7.70E-06
rs72812347:61438785:C:T	10	61438785	T	C	1.426	7.77E-06
rs61849886:23375606:C:T	10	23375606	T	C	0.692	8.13E-06
rs62393909:178220941:T:C	5	178220941	C	T	2.195	8.49E-06
rs16913885	10	61388368	A	G	1.424	8.51E-06
rs4075465:83148925:G:A	16	83148925	G	A	0.7609	8.59E-06
rs1975958	10	23387293	A	G	0.7092	8.65E-06
rs1171655:61446633:C:T	10	61446633	C	T	1.467	9.03E-06
rs35446811:59355925:CA:C	18	59355925	C	CA	0.6164	9.07E-06
rs55755822:59358022:A:ATTAC	18	59358022	ATTAC	A	0.6164	9.11E-06
rs72705319:92309802:C:T	14	92309802	T	C	2.188	9.12E-06
rs1975959:23387323:A:G	10	23387323	G	A	0.7098	9.15E-06
rs138262784:103141583:G:T	12	103141583	T	G	1.734	9.22E-06
rs56106378:30265763:T:A	2	30265763	A	T	1.394	9.45E-06
rs61849888:23376982:A:C	10	23376982	C	A	0.7087	9.46E-06
rs6417253:15135524:C:G	19	15135524	C	G	1.375	9.46E-06
rs6512002:15135471:T:C	19	15135471	T	C	1.375	9.46E-06
rs3793757:23386155:T:G	10	23386155	G	T	0.7105	9.65E-06
rs3838758:23386148:C:CAG	10	23386148	CAG	C	0.7105	9.65E-06
rs55656603:23384238:A:G	10	23384238	G	A	0.7109	1.00E-05
rs56201167:23384152:G:A	10	23384152	A	G	0.7109	1.00E-05
